# Monitoring supports performance in a dual-task paradigm involving a risky decision-making task and a working memory task

**DOI:** 10.3389/fpsyg.2015.00142

**Published:** 2015-02-17

**Authors:** Bettina Gathmann, Johannes Schiebener, Oliver T. Wolf, Matthias Brand

**Affiliations:** ^1^Department of General Psychology: Cognition, University of Duisburg-EssenDuisburg, Germany; ^2^Department of Cognitive Psychology, Ruhr-University BochumBochum, Germany; ^3^Erwin L. Hahn Institute for Magnetic Resonance ImagingEssen, Germany

**Keywords:** decision making under risk, Game of Dice Task, GDT, 2-back task, dual tasking, monitoring, executive functions

## Abstract

Performing two cognitively demanding tasks at the same time is known to decrease performance. The current study investigates the underlying executive functions of a dual-tasking situation involving the simultaneous performance of decision making under explicit risk and a working memory task. It is suggested that making a decision and performing a working memory task at the same time should particularly require monitoring—an executive control process supervising behavior and the state of processing on two tasks. To test the role of a supervisory/monitoring function in such a dual-tasking situation we investigated 122 participants with the Game of Dice Task plus 2-back task (GDT plus 2-back task). This dual task requires participants to make decisions under risk and to perform a 2-back working memory task at the same time. Furthermore, a task measuring a set of several executive functions gathered in the term concept formation (Modified Card Sorting Test, MCST) and the newly developed Balanced Switching Task (BST), measuring monitoring in particular, were used. The results demonstrate that concept formation and monitoring are involved in the simultaneous performance of decision making under risk and a working memory task. In particular, the mediation analysis revealed that BST performance partially mediates the influence of MCST performance on the GDT plus 2-back task. These findings suggest that monitoring is one important subfunction for superior performance in a dual-tasking situation including decision making under risk and a working memory task.

## Introduction

In everyday life people often have to perform two cognitively demanding tasks simultaneously such as making important decisions based on explicit information and maintaining and manipulating information regarding another task. Research has shown that the performance of two cognitively demanding tasks leads to an interference. For example, Baddeley et al. (1984) and Baddeley (1996, 1998) have shown that the performance of two tasks involving the central executive of the working memory (which is supposed to contain several subfunctions, c.f. Baddeley, 1996, 2003) leads to decreased performance in both tasks. The study by Baddeley (1998) investigated the influence of different cognitively demanding tasks on a random generation of numbers, which was assumed to load on the central executive. Results demonstrated that with increase of the additional tasks' cognitive demand the random generation declined. Research focusing on decision making under risk has shown that the simultaneous performance of an additional cognitively demanding task also interferes with the decision-making performance (Starcke et al., 2011; Verbruggen et al., 2012; Pabst et al., 2013; Gathmann et al., 2014a,b). Decision making under risk is repeatedly found to be associated with several executive subfunctions when considering different decision-making tasks (Cambridge Gambling Task, Watkins et al., 2000; Probability-Associated Gambling Task, Bonatti et al., 2008; Balloon Analog Risk Task, Campbell et al., 2013; Columbia Card Task, Buelow, 2014; Game of Dice Task, Schiebener et al., 2014). This is because in decision making under risk the possible consequences of a decision and their probabilities are given descriptively or are at least computable based on the information provided. Thus, in order to make an advantageous decision people can rely on their cognitive functions and establish a decision-making strategy from the very beginning (Brand et al., 2006). For example, participants have to focus on relevant information and to categorize the alternatives. Therefore, the simultaneous performance of a task measuring decision making under risk and an additional cognitively demanding task should interfere with each other. So far, several studies have addressed dual tasking in the field of decision making under risk (Verbruggen et al., 2012; Pabst et al., 2013; Gathmann et al., 2014a,b) and mainly support this assumption: It was demonstrated that making a decision while performing a simple motor control task leads to reduced risky gambling (Verbruggen et al., 2012), while performing a cognitively demanding task (such as the 2-back task) results in an increase of disadvantageous decisions (Starcke et al., 2011; Pabst et al., 2013; Gathmann et al., 2014a). In the current study, we focused on the cognitive processes and the particular involvement of a specific executive function (monitoring) in a dual-task situation in which people are asked to make a decision under risk and to perform a cognitively demanding task (a working memory task) simultaneously.

The studies investigating the influence of an additional cognitive demand on decision making under risk mentioned above mostly used the Game of Dice Task (GDT; Brand et al., 2005) with a simultaneous working memory 2-back task (Starcke et al., 2011; Pabst et al., 2013; Gathmann et al., 2014a,b). The GDT (without additional n-back task) provides the participants with explicit rules and probabilities about a certain amount of gain/loss. In this task, a single die is thrown and participants are asked to bet which number will occur to maximize their starting capital. Participants can either bet on a single number or on combinations of two, three, or four numbers, which are associated with different winning probabilities and amounts of money to gain or lose. In these decision situations executive functions are particularly involved: Participants need to categorize the alternatives according to losses, gains, and probabilities (e.g., Schiebener et al., 2011). Moreover, they can process the feedback in order to adjust the current decision strategy if necessary (Brand et al., 2006, 2009a,b). In a recent study, Schiebener et al. (2014) demonstrated that these executive functions, which they gathered under the term concept formation, as well as monitoring are associated with decision-making performance in the original GDT (without n-back task). Monitoring was understood as the ability to maintain an overall goal in mind while other sub processes are active. For example, while maintaining the task goal in mind (e.g., increase the starting capital) a certain decision strategy might be active (e.g., choosing the low-risk options in a series of five). In order to stick to this strategy monitoring might be necessary to update the current position in this series. Moreover, it appears to be advantageous to check whether the current strategy fits with the overall goal of the task (e.g., by keeping track of the previous success with this strategy). However, Schiebener and colleagues demonstrated that the effects of monitoring and concept formation on decision making under risk are mediated by a general control function. This general control function “stands for the ability to allocate attention according to a task's rules and goals. Thus, general control inhibits the initiation of automatically imposing responses which are not in accordance with the task's rules and goals” (Schiebener et al., 2014 p. 3). In summary, these findings support the assumption that decision making under risk particularly addresses cognitive processes and that monitoring may be one of these cognitive processes (Brand et al., 2006). In the GDT plus 2-back task, the 2-back task is additionally embedded on the left side of the GDT screen. In the 2-back task participants are asked to indicate whether or not the current number presented on the screen is the same as two trials before. It is well known that 2-back task performance is associated with executive functions, such as monitoring, updating, and inhibition (Conway et al., 2005; Owen et al., 2005): Participants have to monitor and update the numbers seen on the screen, have to manipulate their response pattern according to the numbers seen and have to inhibit incorrect reactions. Therefore, it makes sense to assume that in order to do so further executive functions, such as the categorization of stimuli and set maintenance are additionally involved. Participants carrying out the GDT plus 2-back task have the overall aim to perform both tasks as equally and well as possible. The specific executive functions involved in this dual task are still unclear.

Studies actually demonstrated that making advantageous decisions and performing an additional cognitive task simultaneously is associated with various executive functions (Starcke et al., 2011; Pabst et al., 2013; Gathmann et al., 2014a,b). However, all these studies used only one measure of executive functions, for example, the Modified Card Sorting Test (MCST; Nelson, 1976), if any. Therefore, the studies hardly allow for the conclusion that executive subcomponents are crucially involved in GDT plus 2-back task performance.

Consistent with Schiebener et al. (2014) and with the findings regarding the 2-back task (Conway et al., 2005; Owen et al., 2005), we assume an involvement of concept formation (subsuming categorization, set maintaining, feedback processing and rule detection) and monitoring in the simultaneous performance of a decision-making task and an additional working memory task. However, in contrast to a simple decision situation (i.e., GDT solely), we suggest that in order to make a decision and performing a working memory task at the same time requires particularly monitoring. This is because in such complex situations participants need not only to monitor the performance of each single task and the current active process in it, but additionally have to monitor the overall aim of this dual task (i.e., equal performance in both tasks). The question we aimed to address in the current study is which role monitoring plays in a situation in which people have to perform a decision-making task and a working memory task at the same time. The current theoretical view on dual tasking and previous empirical studies suggest a key role of monitoring for dual tasking (as it is demanded from participants performing the GDT plus 2-back task). Theoretically, while concentrating on one task set, the second task set seems to be inhibited (for a review on inhibition in task switching see Koch et al., 2010). Thereby, subjects' attention is narrowed to the prioritized task and consequently shielded from competing distractors (Easterbrook, 1959). However, in order to switch back to the second inhibited task if necessary, this control mechanism simultaneously enables monitoring for potential second-task associated action information (Miller and Cohen, 2001; Plessow et al., 2011). The involvement of monitoring in dual-tasking situations was also postulated by Meyer and Kieras as well as by Norman and Shallice (Norman and Shallice, 1986; Shallice and Burgess, 1991a,b; Meyer and Kieras, 1997a,b). They assumed that a supervisory/monitoring function has to be involved in demanding situations in which an adaption to changing circumstances is necessary (e.g., in dual-tasking situations). An experimental study by D'Esposito et al. (1995) supports this assumption by demonstrating the involvement of cortical areas associated with executive functions during dual tasking, in particular the dorsolateral prefrontal cortex. Findings of further studies which investigated the underlying executive functions in dual tasking indicated that control functions (De Jong, 1995) in particular monitoring and set shifting (Cooper et al., 2012), are involved. However, at this point it should be mentioned that Miyake et al. (2000) did not find any involvement of executive functions, such as shifting, updating, and inhibition in dual tasking.

In sum, several studies have demonstrated that executive functions are important for the simultaneous performance of two cognitive tasks. Furthermore, theory and empirical evidence pointed out a key role of monitoring for dual tasking with cognitively demanding tasks. To test our assumption that monitoring may also be a main contributor to making decisions and performing a cognitively demanding task at the same time, we used again the GDT plus 2-back task in the current study. Each single task (GDT and 2-back task) is associated with various executive functions such as attention, inhibition, updating, comparison of information/categorization of information, set maintenance, and feedback processing (e.g., Conway et al., 2005; Owen et al., 2005; Brand, 2008; Euteneuer et al., 2009; Schiebener et al., 2014). Moreover, these functions were found to be associated with superior performance of a modified version of the GDT plus 2-back task (Gathmann et al., 2014a). One main goal of the GDT plus 2-back task is to work on both tasks simultaneously and to do so equally well. Therefore, especially supervisory/monitoring abilities should be essential for the performance in the GDT plus 2-back task: Each single task and the current progress of it need to be represented in working memory. Thereby, subjects should be able to perform one of the two tasks while the contemporary process of the second task should also be present simultaneously in order to switch back at an appropriate point in time. Still, in order to supervise/monitor performance in a dual-task situation successfully, several executive functions gathered under the term concept formation (e.g., categorization, set maintaining, rule detection, and feedback processing; c.f. Schiebener et al., 2014) should be involved, too. The assumption that some executive functions are interrelated and influence other executive functions is in line with several authors emphasizing that there might be executive functions which are rather basic (e.g., categorization, inhibition, shifting) and others which are of rather higher level (e.g., supervision, monitoring) (Smith and Jonides, 1999; Miyake et al., 2000; Miyake and Friedman, 2012).

Based on the study by Schiebener et al. (2014) we used the newly developed Balanced Switching Task (BST) in order to measure monitoring. Most of the literature assuming an important role of monitoring in dual tasking argue from a rather theoretical perspective (Norman and Shallice, 1986; Shallice and Burgess, 1991a,b; Meyer and Kieras, 1997a,b). Other studies used tasks which measure functions such as set-shifting, updating (Miyake et al., 2000; Cooper et al., 2012), and multitasking (e.g., Manly et al., 2002; Mäntylä, 2013). In these studies authors also discussed the involvement of a supervisory/monitoring function in performing these tasks. For the current study, we used the BST because it was explicitly designed to tap the executive component monitoring. The BST is said to be face valid for tapping monitoring in a relative clear and distinct way (Schiebener et al., 2014). In this task participants are asked to work on four tasks, while only one task at a time can be performed (e.g., to indicate whether the current number is odd or even). All four tasks are similarly difficult and the cognitive effort necessary to perform on them is comparable among the four tasks. The aim of the BST is balanced processing on all four tasks. As a consequence participants have to keep in mind how often they have worked on each task and to remember to switch to the next one or back. The main component for performing all tasks in a balanced way is supposed to be monitoring (Schiebener et al., 2014). We assume that the ability to work on parallel tasks in a balanced way and to monitor the current state of the tasks, which need to be performed, is involved in the performance of the GDT plus 2-back task. Therefore, we used the BST in order to operationalize the described monitoring ability. However, for good monitoring performance as measured by the BST again concept formation should be required. For example, stimuli have to be categorized and compared, and task sets have to be maintained (in order to perform a certain task).

**Figure 1:**
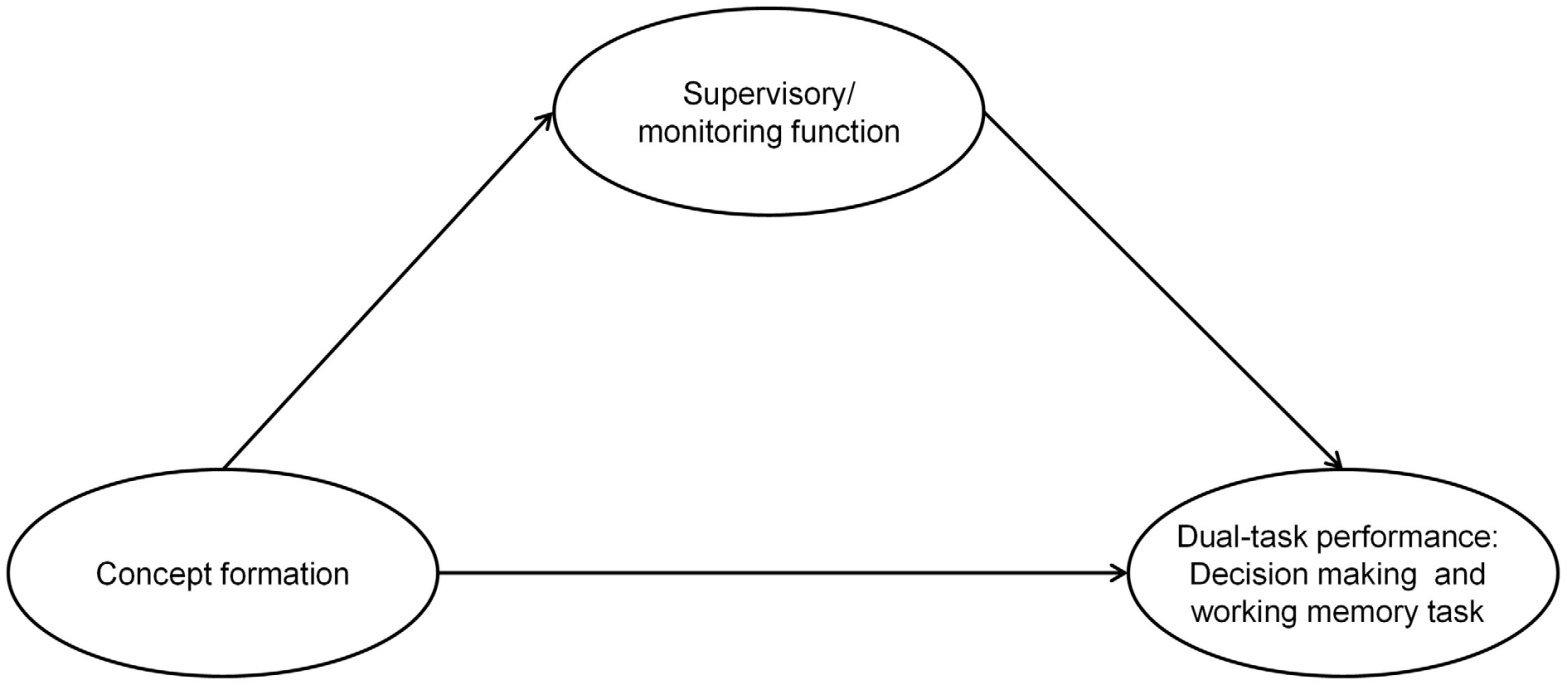
**The theoretical mediation model**. It displays the possible mediation effect of the latent dimension *supervisory/monitoring function* on the relationship between concept formation and the simultaneous performance of decision-making task and working memory task. The arrows represent the assumed direction of the influence of the different variables.

Based on the theoretical considerations above, we assume that concept formation (operationalized by the MCST) and the ability to supervise/monitor performance on different tasks (operationalized by the BST) predict performance in the GDT plus 2-back task. Furthermore, we assume that concept formation is required to constitute the ability to supervise/monitor working on different tasks (BST). Moreover, this supervision/monitoring ability may affect performance in the GDT plus 2-back task. In other words, we expect that the effect of concept formation on the GDT plus 2-back task performance is mediated by monitoring functions. However, even though a supervisory/monitoring function appears to be important to perform well on the GDT plus 2-back task, it is likely that concept formation measured by the MCST is additionally involved in the GDT plus 2-back performance. Therefore, only a partial mediation is expected (see Figure 1).

## Material and methods

### Participants

Overall, we examined 122 right handed participants (mean age: 31.06, *SD* = 13.07 years; 62 females). The participants were students of the University of Duisburg-Essen as well as their relatives and friends. The participation was voluntary and the study lasted an hour for which the participants received either credit points or a financial compensation of €10. As determined by a self-report questionnaire none of them had a history of neurological or psychiatric diseases. All participants demonstrated average estimated IQ performance, measured by the subtest four (reasoning) of the German intelligence test battery *Leistungsprüfsystem* (LPS; Horn, 1983), *M* = 117.03, *SD* = 11.95. Participants with an age higher than 50 were screened for dementia with the DemTect (Kalbe et al., 2004). None of them had a score lower than 13, indicating no signs of mild cognitive impairment or dementia. All participants gave written informed consent. The study was approved by the local ethics committee.

### Instruments

In reference to the theoretical model postulated in the introduction, the different variables were operationalized as follows: As a measure of a dual-tasking situation involving the simultaneous performance of decision making under explicit risk and a working memory task the GDT plus a parallel working memory task (2-back) was used (c.f. Starcke et al., 2011). In order to operationalize concept formation the MCST was applied. To assess monitoring the newly developed BST (Schiebener et al., 2014) was used. All tasks are now described in detail.

#### Dual tasking: Game of Dice Task (GDT) plus a parallel working memory task (2-back)

**Figure 2:**
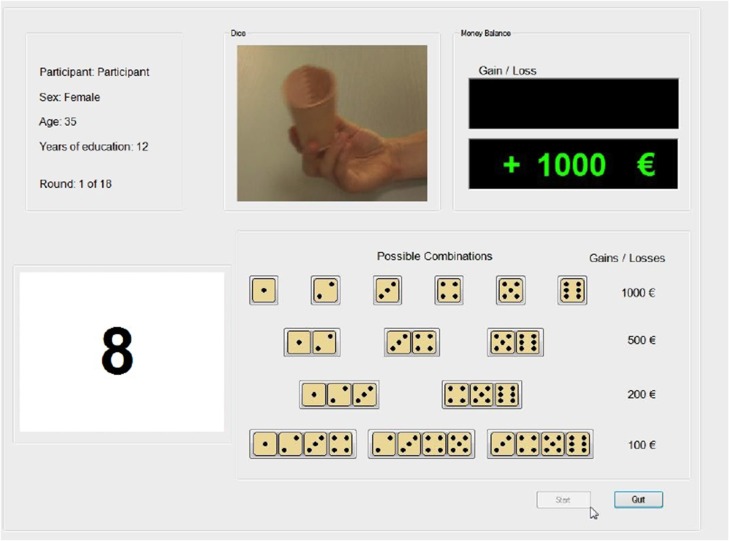
**Game of Dice Task plus 2-back task**. On the right side of the screen, participants work on the Game of Dice Task (GDT) by betting which number will be thrown next. On the left side of the GDT interface, participants must solve the 2-back task. Here, they need to continuously monitor the numbers presented and have to indicate whether the current number was presented two trials before or not, by keyboard input.

The same dual-task paradigm used in the study by Starcke et al. (2011) was applied in the present study. It included a decision-making task with explicit and stable rules—the GDT (Brand et al., 2005)—and a parallel working memory 2-back task. Both tasks were presented on the same computer screen: The 2-back task was embedded into the GDT interface, such that the 2-back task was presented on the left side of the screen while the GDT was on the right side (see Figure 2). In order to work on both tasks simultaneously, participants had to use their left hand for the 2-back task and the right hand for the GDT. The participants were told to perform on both tasks to the best of their abilities and to put equal effort into working on each task.

The GDT is a computerized task which is often used to operationalize decision making under risk (c.f. Gleichgerrcht et al., 2010). In this task participants aim to maximize the fictitious starting capital of €1000. During the 18 trials in which one virtual die is thrown, participants are asked to guess which number will be thrown next. In order to comply with the aim participants are asked to bet on one single number or on a combination of two, three, or four numbers by clicking the respective button using the computer mouse. They win if the chosen number or one number out of the chosen combinations of numbers is thrown, otherwise they lose. The options as well as the possible gains and losses are permanently shown on the screen. Each offered option is associated with different winning probabilities. When choosing one single number (e.g., six) the winning probability is 16.67% to gain €1000. If one of the other five numbers is thrown (one, two, three, four, or five) €1000 will be lost. Choosing the combination of two numbers (e.g., three and four), leads to a gain of €500 with a probability of 33.33%. However, if one of the other four numbers is thrown (one, two, five, or six) participants will lose €500. The combination of three numbers (e.g., one, two, and three) provides a gain of €200 with a winning probability of 50%. If one of the other three numbers is thrown (four, five, or six) they will lose the same amount. With a winning probability of 66.67% the choice of the combination of four numbers (e.g., three, four, five, and six) leads to a gain of €100 and if one of the other two numbers is thrown (one or two) participants lose €100. Following each decision participants receive visual feedback about the amount of gain (colored green) or loss (colored red). Furthermore, the current balance and the remaining rounds are also permanently shown on the screen. In total, the options can be categorized into advantageous/low-risk decisions (combinations of three and four numbers with a winning probability of 50% and higher) and disadvantageous/high-risk decisions (one single number and combinations of two numbers with a winning probability of less than 34%). Choosing the advantageous options all the time would statistically lead to a positive outcome in the long run, given the starting capital of €1000. Therefore, the combination of three numbers is also classified as low risk in accordance with other studies (e.g., Brand et al., 2009b; Bayard et al., 2011).

The working memory 2-back task has to be performed simultaneously to the GDT. In the small window on the left side of the GDT interface participants are shown randomized numbers between 0 and 9 in succession. They are asked to indicate for each presented number whether or not it is identical with the number two trials before. The digits are displayed for 500 ms with an inter-stimulus interval of 2750 ms. In the time frame of 500 ms participants are asked to indicate their answer by pressing one of two keyboard buttons (e.g., “c” for yes, digits are identical and “x” for no, digits are not identical). Answers are distinguished between correct reactions, false reactions, and skips (i.e., if no answer is given during a trial). Immediately after indicating their answer and before the next stimulus is presented, participants receive visual feedback whether their answer was correct (green check) or false/omitted (red cross). The target stimuli, the same stimulus as two trials before, are displayed randomly with a probability of 33% (adapted from Schoofs et al., 2008).

In order to analyze the performance in the GDT, several scores were computed:

1. Expected final capital: The theoretically expected final outcome, considering all decisions of the participants and the expected value of their decisions (expected final capital = starting capital + the sum of the expected values of all choices).
2. Actual final capital: Indicates the actual capital at the end of the task.
3. Net Score: A positive net score indicates advantageous decision-making performance (net score = low-risk decisions − high-risk decisions).
4. Percentages of low-risk decisions.

Moreover, we calculated a standardized score of the expected final capital in order to be able to compute an overall score of the GDT plus 2-back task. Therefore, we transformed the variable expected final capital into a scale with zero being the lowest possible capital (by adding the theoretically lowest expected final capital to the expected final capital of each participant). The resulting value was then transformed into percentages. In order to analyze the performance of the 2-back task we used percentages of correct reactions (i.e., correct identification and correct refusal as a target digit) as main measure. For the analysis of the *overall performance* of the GDT plus 2-back task, the mean of the standardized, expected final capital (in percent) in the GDT and the percentages of the correct answers in the 2-back task was computed. This overall performance of the GDT plus 2-back task was used as a main measure of the dual-tasking performance and included in the structural equation model (SEM). However, we had to take into account that the net score is the commonly used score of the GDT. Therefore, we additionally calculated an overall score which includes a similar score as the GDT net score: The mean of the low-risk decisions in percent and the percentage of correct answers in the 2-back task.

#### Concept formation: Modified Card Sorting Test (MCST)

The Modified Card Sorting Test (MCST; Nelson, 1976) is mostly used to measure executive functioning in general. This is because solving the test requires several executive abilities, involving categorization, set maintenance, rule detection, and the ability to use feedback. In this computerized test participants are asked to sort 48 cards (one at a time) onto one of four card decks presented on the screen according to a particular predetermined rule which is unknown to the participants. The symbols on the cards differ with respect to shape, color, and amount of symbols. Accordingly, the cards can be sorted in three ways: by the shape, the color, and the number of the stimuli on the cards. Participants have to figure out which rule to apply through trial-and-error using the provided feedback (right or wrong). The rule changes after six consecutive correct responses.

Although the MCST has been found to load on set shifting/cognitive flexibility and inhibition (see e.g., Miyake et al., 2000), we assume in line with Schiebener et al. (2014) that the three main abilities required for the MCST are categorization, rule detection, and set maintenance. This is because participants need to use feedback to identify the current card sorting rule and have to apply the rule according to different card symbols and categories.

The number of perseverative errors was used as a main measure in the current SEM. Perseverative errors occur when the participant continues to sort cards according to the previous rule though it had been indicated that the rule had changed, indicating problems in using feedback and categorizing correctly (De Zubicaray and Ashton, 1996).

#### Supervisory/monitoring function: Balanced Switching Task

The BST (Schiebener et al., 2014) is based on the voluntary task switching paradigm used by Arrington and Logan (2004) and was developed to measure monitoring abilities. In this computerized task participants have to deal with four tasks, which they are asked to perform with equal effort. To comply with the aim participants have to voluntarily switch between tasks. The BST consists of two sets of stimuli: Set A contains numbers from “01” to “99” and set B contains abstract geometric shapes which are diagonally hatched. In each set participants can work on two tasks respectively. Responses are made on a QWERTZ-Keyboard using the keys “d” (left middle finger) and “f” (left index finger) as well as “j” (right index finger) and “k” (right middle finger). In set A, task 1 requires to indicate whether the currently presented number is even (“d”) or odd (“f”). In task 2, participants are asked to indicate whether the presented number is smaller (“j”) or greater (“k”) than the number 50. To avoid ambiguity, the number 50 itself was not part of the stimuli. In set B, task 1 requires to indicate whether the hatching of the currently presented shape goes left (from the right lower corner of the shape to the left upper corner) by pressing “d” or right (from the left lower corner of the shape to the right upper corner) by pressing “f.” In task 2 of set B, participants are asked to indicate whether the presented shape is oriented vertically (“j”) or horizontally (“k”). To switch between set A and set B participants were told to press the spacebar. Switching within one set between the two tasks can easily be done by using different keys: “d” and “f” are associated with task 1 of each set and “j” and “k” are associated with task 2 of each set. Only one stimulus is presented at a time and participants have to process only one out of the four tasks with each presented stimulus.

Before the task starts participants complete a practice trial in which the examiner makes sure that the participants learn how to work on the tasks. Participants are informed that the goals of the paradigm are to perform all four tasks in a balanced fashion, to classify the stimuli to the best of their ability, and to execute as many stimuli as possible in a given time. Moreover, they are informed that the switch between the two sets is associated with a certain time frame (1250 ms) in which participants cannot work on any task. This indicates that participants would have less overall processing time for the tasks if they switched between sets too often. This rule should increase the load on monitoring, because it is assumed that this rule motivate to stay with one task for a longer time. To stay for a longer time with one task should increase the cognitive effort of keeping track of how long and how often they have worked on the other tasks before. Moreover, it should increase the effort of remembering that further switches need to be made in order to stick with the overall goal to work on all four tasks equally. The subjects are neither informed about the overall duration of the paradigm nor about the stimulus presentation times. Therefore, participants are not able to estimate how much time they will have to work on each task to achieve the best balance between all tasks. In total, the task consists of two blocks of 4 min each. A stimulus is presented until the participant responds, but maximally for 1000 ms. The inter-stimulus interval is 500 ms, a switch between set A and set B costs 1250 ms of the overall time. In order to reduce potential effects of vigilance and fatigue, a short break of 60 s was set between the blocks. In this break, as well as after the second block, participants receive feedback about the equability of the tasks performances in percent, the total accuracy in percent, and the number of stimuli the participant responded to correctly.

In order to evaluate the supervisory/monitoring performance postulated in the SEM, the so called *deviation score* was computed for each block separately and across both blocks. The deviation score provides information about the deviation from the optimal equal performance calculated for each participant. The deviation score for a single block was calculated as follows:

$$\sqrt{\frac{\begin{array}{l} {\left(\text{task }1\text{ Set A }-\;0.25\right)}^{2}+{\left(\text{task }2\text{ Set A }-\;0.25\right)}^{2}\\ +{\left(\text{task }1\text{ Set B }-0.25\right)}^{2}+{\left(\text{task }2\text{ Set B}-0.25\right)}^{2} \end{array}}{4}}$$

For each task the amount of presented stimuli in percent was calculated (e.g., presented stimuli of task 1 in set A divided by presented stimuli of block 1). In the formula above, this is displayed by *task 1 set A, task 2 set A, task 1set B*, and *task 2 set B*. From each of these results the optimal value of equal performance (25%) was subtracted, and the outcome was squared. We calculated the mean from this equation and then calculated the square root of the mean. A deviation score of 0% testifies to equal adaptation of all four tasks (i.e., perfect performance). A deviation of 43% indicates that the participant had only performed one task out of four (i.e., worst performance).

### Statistical analyses

For the statistical standard analyses IBM SPSS Statistics software for Windows (Release 19.0; April 18, 2011; SPSS Inc. IBM, Chicago) was used. To test for zero-order relationships between two variables Pearson correlations were calculated. In order to test the hypothesized mediation model, SEM analysis was done using Mplus 6 (Muthén and Muthén, 2011). For this the maximum likelihood parameter estimation was applied. We had directed mediation hypotheses. Therefore, one-sided testing is advisable. Mplus always tests two-sided which we correct by using *p* ≤ 0.100 and a CI of 90% as significance thresholds for the mediation analyses. There were no missing data.

The evaluations of the model fits were done by applying standard criteria (Hu and Bentler, 1995, 1998, 1999). The following fit indices were used: χ^2^ test (non-significant values indicate that the data do not significantly differ from the model), χ^2^/*df* (values between 0.00 and 2.00 indicate a good fit), root mean square of approximation (RMSEA; “test of close fit”; a value between 0.00 and 0.05 with a significance value between 0.10 and 1.00 indicates a good fit), standardized root mean square residual (SRMR; values between 0.00 and 0.05 indicate a good fit), comparative fit index (a value between 0.97 and 1.00 indicates a good fit), Tucker-Lewis Index (TLI; values between 0.97 and 1.00 indicate a good fit). Due to the fact that the TLI is not standardized, sometimes the values can be outside the range of 0.00–1.00 (Schermelleh-Engel et al., 2003). According to Baron and Kenny (1986) it is required that all variables included in the mediation correlate with each other. Therefore, we included such analyses for the assumed theoretical model. Given two models, the Bayesian information criterion (BIC) is used for model selection. In line with Kass and Raftery (1995) the model with the smaller BIC value is preferred. A difference of the BIC scores >10 demonstrates a very strong validity that the one with the smaller BIC value is the best fitting model.

## Results

### Descriptive data of task performance

Table 1 shows the mean performances of the participants in the different tasks. Compared to studies which used the original version of the GDT in healthy subjects demonstrating a net score around 10 (c.f. Brand, 2008; Brand et al., 2008, 2009a), the net score of the GDT version used here was lower on a descriptive level. However, it was similar to the net score found in the study by Starcke et al. (2011) that previously used the GDT plus 2-back task. The correct responses in percent of the 2-back task performed simultaneously to the GDT were lower on a descriptive level than in studies where participants had to perform only the 2-back task (Knops et al., 2006; Keeser et al., 2011). The performance in the MCST was in a normal range (Lineweaver et al., 1999). The values of the BST were on a descriptive level similar to the values in the study by Schiebener et al. (2014). They indicate that the instructions of the tasks were understood and implemented. Moreover, the difficulty of the task seemed to be adequate: The mean of the deviation was about 10% indicating that on average the participants performed well on the tasks. Yet, there was variance in the performance (see range and standard deviation in Table 1) which suggests that some participants had difficulties in equal performance of the four tasks while others did not.

**Table 1:** **Descriptive values of task performances of the sample**.

**Tests**	**Range**	**M**	**SD**
**GDT**			
Net score^a^	−18–18	7.03	9.57
Low-risk decisions^b^	0–100	69.54	26.57
Final capital^c^	−13300.00–3000.00	−704.10	2649.54
Expected total capital^c^	−8832.50–1600.12	−554.32	2223.53
Standardized, expected total capital^b^	17.19–100	82.90	17.65
**2-BACK TASK**			
Correct responses^b^	9.43–89.04	57.88	18.34
**GDT PLUS 2-BACK TASK**			
Mean of correct responses in the 2-back task and the standardized expected total capital of the GDT	26.79–94.08	70.39	14.37
**MCST**			
Perseverative errors^c^	0–8	1.13	1.78
**BST**			
Deviation score block 1^d^	0.00–0.43	0.10	0.10
Deviation score block 2^d^	0.00–0.43	0.09	0.09
Deviation score (block 1 and 2)^d^	0.00–0.43	0.08	0.09

*GDT, Game of Dice Task; MCST, Modified Card Sorting Test; BST, Balanced Switching Task*.

a *Low-risk decisions minus high-risk decisions*.

b *Percentages*.

c *Raw score*.

d *Relative frequencies*.

### Correlations between GDT plus 2-back and executive functions

Table 2 demonstrates that variables used for executive measurements (MCST and BST) in the theoretical model correlated significantly with the GDT plus 2-back task. We additionally investigated the relationship between each single task score and the executive measurements. These analyses revealed that the score of the 2-back task (correct responses in percent) significantly correlated with the MCST and the BST. However, there was neither a significant correlation between the GDT score (standardized, expected total capital in percent) and the MCST nor the GDT score and the BST.

**Table 2:** **Correlations between the Game of Dice Task (GDT) plus 2-back task and executive functions**.

		**1**	**2**	**3**	**4**	**5**	**6**
1	GDT plus 2-back task^a^	–	–	–	–	–	–
2	MCST^b^	−0.299^***^	–	–	–	–	–
3	BST (block 1)^c^	−0.272^**^	0.254^**^	–	–	–	–
4	BST (block 2)^c^	−0.240^**^	0.237^**^	0.784^***^	–	–	–
**SUB SCORES**							
5	GDT^d^	0.790^***^	−0.122	−0.097	0.052	–	–
6	2-back task^e^	0.807^***^	−0.352^***^	−0.333^***^	−0.427^***^	0.276^**^	–

*MCST, Modified Card Sorting Test; BST, Balanced Switching Task*.

a *Mean of correct responses in the 2-back task and standardized expected total capital (percentages)*.

b *Frequency of perseverative errors (raw score)*.

c *Deviation score (relative frequencies)*.

d *Standardized expected total capital (percentages)*.

e *Correct responses in percent*.

** *p ≤ 0.010*,

*** *p ≤ 0.001*.

### The latent dimension

The high β coefficients of the two manifest variables of the BST (deviation score block 1: β = 0.930, *SE* = 0.09, *p* ≤ 0.001; deviation score block 2: β = 0.843, *SE* = 0.08, *p* ≤ 0.001) revealed that the latent dimension *supervisory/monitoring function* seems to be adequately modeled. Moreover, this is supported by the significant correlation, *p* ≤ 0.001, of the two variables with a high effect size (see Table 2).

### Full SEM

Testing the proposed model with the overall performance in the GDT plus 2-back task as endogenous variable revealed a good fit with the data. The χ^2^ test was not significant, indicating that the data do not differ significantly from the model, χ^2^ = 0.041, *df* = 1, *p* = 0.840. The ratio of χ^2^/*df* was below 2.00, the RMSEA had a value <0.01 with *p* = 0.862, the SRMR was 0.002, the CFI was 1.00 and the TLI = 1.042.

#### The pathways of the full model

**Figure 3:**
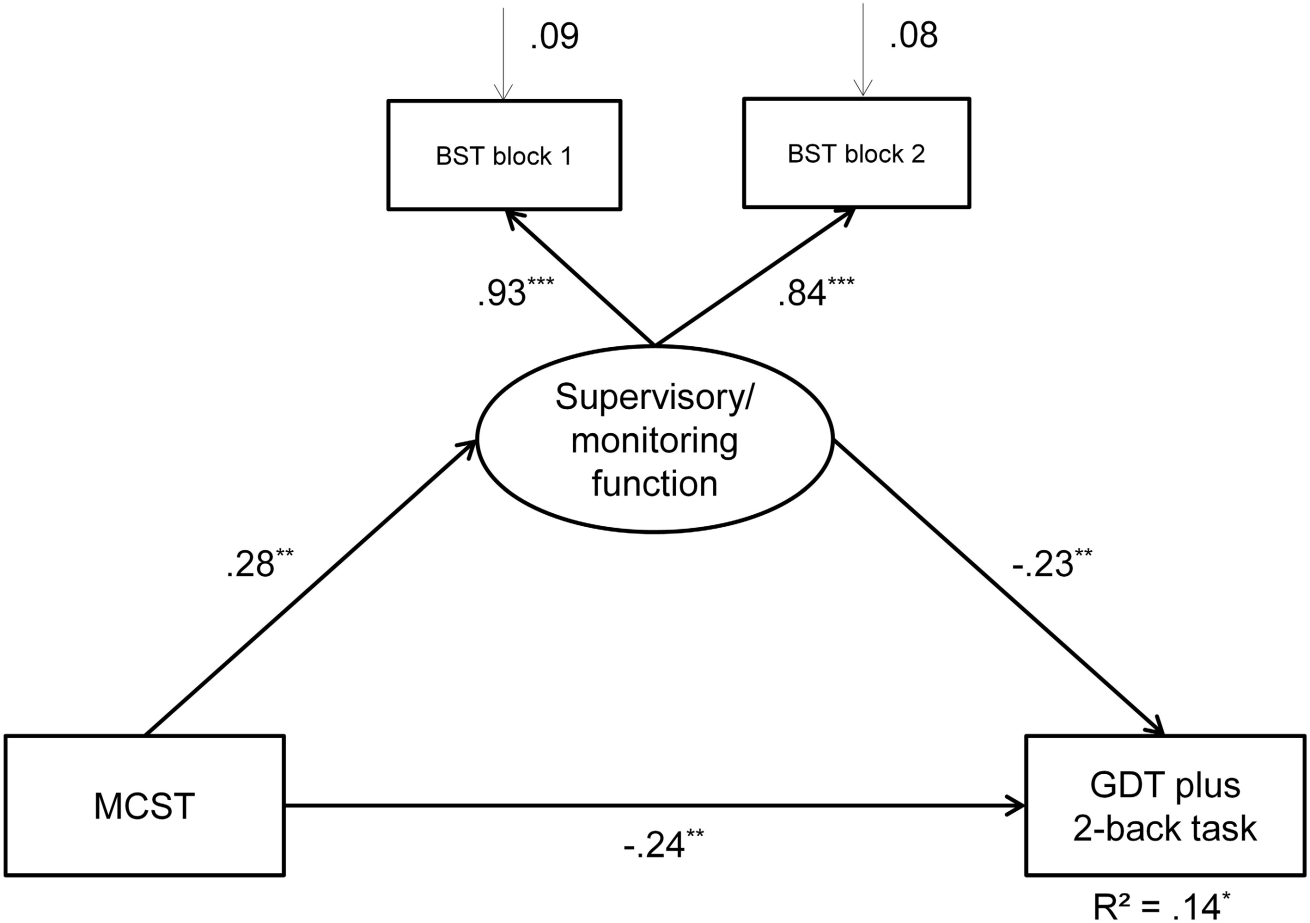
**The full structural equation model**. The oval shape indicates the latent dimension while the rectangular shapes indicate the manifest variables. Bold arrows indicate direct effects while the non-bold arrows display errors. ^***^p ≤ 0.001, ^**^p ≤ 0.001, ^*^p ≤ 0.050. BST, Balanced Switching Task, measures the supervisory/monitoring function; MCST, Modified Card Sorting Test, measures concept formation; GDT, Game of Dice Task, measure for decision making under risk; 2-back task, working memory task.

Figure 3 demonstrates that in total 14% of the variance of the GDT plus 2-back task could be explained significantly by the model, *SE* = 0.06, *p* = 0.021. Monitoring, measured by the BST, as well as concept formation, measured by the MCST, explained the GDT plus 2-back variance significantly, *SE*'s ≤ 0.09, *p*'s ≤ 0.014. The β coefficients were negative because good performances in the MCST and BST are indicated by low values.

### Mediation analysis

In order to analyze whether the effect of concept formation on dual-task performance (GDT plus 2-back task) is mediated by monitoring a mediation analysis was computed. Using the corrected significance thresholds (see statistical analysis section, *p* = 0.100, CI 90%) the indirect effect from MCST via the latent dimension *supervisory/monitoring function* on the GDT plus 2-back task was significant, β = −0.062, *p* = 0.059. The direct effect from concept formation on GDT plus 2-back task was significant as well, β = −0.237, *p* = 0.006. We additionally ensured these finding by testing the significance of the indirect and direct effect using bias-corrected bootstrapping procedure. These effects were computed for 10,000 bootstrapped samples. The indirect effect was again significant, *p* = 0.091, CI 90%: [−0.122, −0.002]. The direct effect was significant as well, *p* = 0.002, CI 99%: [−0.436, −0.039]. Thus, there was a partial mediation from concept formation over of the *supervisory*/*monitoring function* on the GDT plus 2-back performance (see Figure 3).

We calculated further additional analyses which support the reported main results. Information regarding these additional findings can be found in the appendix of the manuscript.

## Discussion

The current findings give insight to a part of the underlying cognitive processes involved in the simultaneous performance of decision making under risk and a working memory task. As presumed in the theoretical model concept formation (operationalized by the MCST) and monitoring (operationalized by the BST) are involved in the simultaneous performance of decision making under risk and a working memory task (operationalized by the GDT plus 2-back task). In more detail, it was shown that BST performance partially mediates the influence of MCST performance on the GDT plus 2-back task. This may be due to the fact that concept formation (operationalized by the MCST) is a component of monitoring (operationalized by the BST).

Overall, the assumed involvement of executive functions in the simultaneous performance of decision making under risk and a working memory task is in line with previous studies (Starcke et al., 2011; Pabst et al., 2013; Gathmann et al., 2014a,b). Moreover, the current findings are in agreement with studies which argue that a supervisory/monitoring function plays a key role in dual-tasking processes in general (e.g., De Jong, 1995; Meyer and Kieras, 1997a,b; Cooper et al., 2012). However, this is the first study suggesting a crucial role of a specific executive function (the supervisory/monitoring function) in a dual-tasking situation in which a decision has to be made and an additional working memory task has to be performed at the same time. The involvement of concept formation in a supervisory/monitoring function might be seen in analogy to Smith and Jonides (1999) who also postulated that there are executive functions such as attention, inhibition, and task management, which are the most basic functions and fundamental ingredients of higher executive functions (e.g., planning, monitoring).

The finding that the supervisory/monitoring function appears to play a crucial role in the simultaneous performance of a decision-making task and a working memory task supports the assumption of several studies that performing both tasks simultaneously cannot be done automatically but requires substantial cognitive control (Starcke et al., 2011; Pabst et al., 2013; Gathmann et al., 2014a,b). According to Stuss et al. (1995) and Shallice et al. (2008) situations which require cognitive control involve besides monitoring further executive functions, such as energization of schemata, task-setting (i.e., adjusting task performance from a novel state to a rather routinized one), and control of if-then logical processes (i.e., applying task rules). These functions might account for further variance in the GDT plus 2-back task, given that in the current study monitoring (operationalized by the BST) only partially mediates the influence of concept formation (measured by the MCST). Moreover, these further executive functions may also represent additional mediators or even moderators. Therefore, they may be addressed in future examinations in order to better understand the interaction of cognitive functions involved in dual-tasking situations in which a decision has to be made and a working memory task has to be performed simultaneously.

Regarding the correlational analyses, which are separated for the GDT as well as the 2-back score of the GDT plus 2-back task with executive functions, it appears that particularly the 2-back task score correlates with concept formation (measured by the MCST) and monitoring (measured by the BST). In contrast, there was no significant relationship between the GDT score and executive functions measured by the BST and MCST. This seems to be in contrast to studies demonstrating the involvement of these functions in the GDT performed solely (e.g., Brand et al., 2007, 2009a; Schiebener et al., 2014). However, the correlation between executive functions and GDT performance when performed without an additional working memory task cannot be compared with the correlation between executive functions and the GDT score of the GDT plus 2-back task. This is because it was found that performing an additional working memory task and the GDT simultaneously influences GDT performance significantly. In other words, decision making differs when performed solely or with a simultaneous 2-back task. The same might account for the 2-back task, but there is so far no study investigating this assumption. However, comparison with previous studies demonstrate on a descriptive level that 2-back task performance without a simultaneous decision-making task was higher than in the current study (59%). For example, Keeser et al. (2011) using a 2-back task with digits solely reported an accuracy score of 66% (mean accuracy rate: 0.66). In another study the accuracy score was 88% (Knops et al., 2006). Furthermore, the performance on the dual task (GDT plus 2-back task) requires more than to simply sum up the processes involved in making decisions in the GDT solely and in responding to the 2-back task solely. It requires additional processes which are responsible for ensuring that both tasks have to be solved at the same time. Thus, based on the correlations it is not possible to conclude that concept formation and monitoring are important for the 2-back task but not for the GDT, because the dual-tasking situation is different from simply adding the 2-back task performance and the GDT performance. Moreover, it was found that GDT performance is associated with working memory (Drechsler et al., 2007; Schiebener et al., 2013). Thus, simultaneous performance of a working memory task in parallel with the GDT might absorb the amount of working memory in the GDT (as part of the GDT plus 2-back task), leading to non-significant correlations between the GDT score of the GDT plus 2-back task and executive functions measured by the MCST and BST. Furthermore, the correlation between the 2-back task score and the GDT score demonstrates a relationship between the two subtasks when belonging to one dual task (see also Starcke et al., 2011). Therefore, the correlations between the sub scores and the executive functions have to be treated with caution and are hardly comparable with the correlation between executive functions and each task when performed solely.

The current results point in the direction that the role of certain executive functions changes when the decision situation is more complex: Performing only a decision-making task (GDT) particularly involves a general control function which mediates the influence of monitoring and concept formation on decision making under risk (Schiebener et al., 2014). In contrast, when making a decision and performing an additional working memory task simultaneously (GDT plus 2-back task), it appears that monitoring (operationalized by the BST) becomes more important and at least partially mediates the effect of concept formation (measured by the MCST) on the dual-task performance (i.e., GDT plus 2-back task). At this point it has to be mentioned that we did not measure general control which might also be involved in the dual-tasking situation and explain further variance. However, our finding of the crucial role of monitoring in dual-tasking situations involving a decision-making task and a working memory task might further enlighten the results by Pabst et al. (2013) and Gathmann et al. (2014b). They found that after stress induction participants demonstrated no impairments in task performance when working on a decision-making task and a working memory task at the same time. This is in contrast to the findings by Starcke et al. (2011) which demonstrated impairments at least in decision-making performance when performing a working memory task simultaneously but without prior stress induction. Pabst et al. (2013) and Gathmann et al. (2014b) argued that the unimpaired task performance might be due to a serial-to-parallel processing shift triggered by stress, which allows the parallel instead of serial performance of two tasks, resulting in preserved task performance (for a detailed discussion about this shift be referred to Pabst et al. (2013) and Gathmann et al. (2014b). Yet, little was known about the particular executive functions involved in this process. Gathmann et al. (2014b) demonstrated an increased activation in the anterior prefrontal cortex, which is besides parallel processing associated with executive functions (Koechlin and Hyafil, 2007). Due to the fact that the supervisory/monitoring function is associated with activity in the frontal lobes (c.f. Stuss et al., 1995), it can be assumed that this function was also increased during the stressful situation in the study by Gathmann et al. (2014b). This might have facilitated the simultaneous performance of the decision-making task and the working memory task. An increased supervisory/monitoring function in such demanding situations enables not only the activation of the relevant task set and the monitoring for potential second-task associated action information (Miller and Cohen, 2001; Plessow et al., 2011), but also ensures that there are few incorrect responses (Stuss et al., 1995). Thus, an increased supervisory/monitoring function may have the potential to provide unimpaired task performance when performing a decision-making task and a working memory task simultaneously in a stressful situation. Taken together, and with regard to the current decision-making research it appears that executive functions are differentially demanded in simple compared to complex decision situations: Monitoring becomes more crucial in situations in which one has to make a decision and to perform a working memory task simultaneously.

### Limitations and future studies

At this point, some limitations of the current study have to be mentioned. First of all, we did not measure further executive functions in order to analyze their influence on the joint performance of the GDT plus 2-back task. Yet, as mentioned above, there might be further executive functions representing a potential mediator, for example general control (Schiebener et al., 2014), energization of schemata, or task setting (Shallice et al., 2008). Therefore, we can only assume which further executive functions might be involved. Secondly, the assumption that the BST measures monitoring/supervisory functions is based on its conceptualization and structure rather than empirical evidence. This means that beyond face validity of the task, the psychometric properties including convergent and divergent validity should be tested in future studies. However, in terms of face validity, we believe that it is reasonable that the task loads on the supervisory/monitoring function because participants have to supervise consequently how much time they had already spend on each subtask. Moreover, while participants are working on one subtask, they have to keep in mind that they have to switch to the other three subtasks to obey the aim of balanced performance. According to Shallice et al. (2008) such an ability represents monitoring. To test this assumption empirically, future studies should investigate convergent and divergent validities by correlating performance on the BST with performances in other executive functioning tests. Moreover, strategies used in the BST and GDT plus 2-back task should be investigated in more detail. The combination of the investigation of strategies and executive functions their involvement in these tasks might on the one hand help to better understand which functions/strategies are important for dual tasking/multitasking, on the other hand, it might explain how people try to perform all tasks equally and thereby give a deeper insight into the understanding of monitoring and the cognitive functions necessary for superior monitoring.

Investigating the underlying cognitive processes of such dual tasks in future studies appears to be a recent and important topic: In everyday life people commonly have to perform two tasks in parallel (Wu et al., 2013) or have to consume more than one item or stream of content at a time (Ophir et al., 2009). Some studies demonstrated that after a training session laboratory dual-task performance of participants increases (e.g., Bherer et al., 2005; Liepelt et al., 2011). Thus, gathering knowledge about which executive functions are involved in the simultaneous performance or processing of information/tasks could help to develop dual-task trainings with more practical application in everyday life to prevent dual-task performance from decreasing.

## Conclusion

We conclude that a dual-tasking situation in which a decision has to be made and a working memory task (operationalized by the GDT plus 2-back task) has to be performed at the same time involves concept formation (as measured by the MCST). Additionally, the influence of concept formation appears to be partially mediated by the supervisory/monitoring function (operationalized by the BST), which is nonetheless still influenced directly by concept formation. It appears that monitoring and concept formation are important subfunction for superior performance in a dual-tasking situation including decision making under risk and a working memory task.

### Conflict of interest statement

The authors declare that the research was conducted in the absence of any commercial or financial relationships that could be construed as a potential conflict of interest.

## Appendix

In order to better understand the underlying mechanisms of decision making when performing an additional working memory task, we tested a further possible control model. To test whether monitoring might be the predictor and concept formation the mediator, we computed a model changing the two variables accordingly. The model revealed the same model fit indices as the theoretical model: The χ^2^ test was not significant, indicating that the data do not differ significantly from the model, χ^2^ = 0.041, df = 1, *p* = 0.840. The ratio of χ^2^/df was below 2.00, the RMSEA had a value <0.01 with *p* = 0.862, the SRMR was 0.002, the CFI was 1.00 and the TLI = 1.042. Using the corrected significance thresholds (see statistical analysis section, *p* = 0.100, CI 90%) the indirect effect from the latent dimension via the MCST on GDT plus 2-back task was significant, β = −0.065, *SE* = 0.03 *p* = 0.039. The direct effect was significant as well, β = −0.225, *SE* = 0.09, *p* = 0.014. However, the difference between the two BIC values was much higher than 10 which indicates a better fit for the theoretical model postulated in Figure 2 (BIC theoretical model: 441.680, BIC control model: 937.366).

Due to the fact that in most studies the net score is used for analyzing GDT performance, we also calculated a performance score of the GDT plus 2-back task including the low-risk decisions in percent: Mean of correct responses in the 2-back and the low-risk decisions in percent. The model revealed again a good fit with the data, χ^2^ = 0.71, df = 1, *p* = 0.400. The ratio of χ^2^/df was below 2.00, the RMSEA had a value <0.01 with *p* = 0.468, the SRMR was 0.01, the CFI was 1.00 and the TLI = 1.01, providing again a partial mediation of monitoring on dual-task performance: Using the corrected significance threshold (*p* = 0.100, CI: 90%) the indirect effect was significant, β = −0.050, *SE* = 0.03, *p* = 0.096, as well as the direct effect: β = −0.201, *SE* = 0.09, *p* = 0.023. However, the difference between the two BIC values was much higher than 10 which indicates a better fit for the theoretical model postulated in Figure 2 (BIC theoretical model: 441.680, BIC control model with the mean of correct responses in the 2-back and the low-risk decisions in percent as dependent variable: 502.980). A similar pattern was found when testing again the assumed theoretical model (see Figure 1), but this time changing the mediator variable into a manifest variable instead of a latent dimension (the overall deviation score without discriminating between block 1 and block 2). Given that this model was calculated on manifest level, the model fit was good: RMSEA had a value <0.001, CFI and TFI had both a value of 1.00, and the value of the SRMR was <0.001. Using again the corrected significance threshold (*p* = 0.100, CI: 90%) the indirect effect from the MCST via the BST on GDT plus 2-back task performance was significant, β = −0.049, *SE* = 0.03, *p* = 0.074. The direct effect from MCST on GDT plus 2-back task was as well significant, β = −0.251, *SE* = 0.09, *p* = 0.003. The difference between the two BIC values was much higher than 10 which indicates a better fit for the theoretical model postulated in Figure 2 (BIC theoretical model: 441.680, BIC control model with an overall deviation score: 750.002).

In summary, all these control models supported the idea that monitoring mediates the effect of concept formation on GDT plus 2-back. Furthermore, even when using other variables for representing BST or GDT plus 2-back performance, all main results remain stable. However, compared to the assumed theoretical model in Figure 2 theses models appear to fit less good to the data.
